# Antimicrobial resistance and genomic characterization of *Salmonella* Dublin isolates in cattle from the United States

**DOI:** 10.1371/journal.pone.0249617

**Published:** 2021-09-21

**Authors:** Mariela E. Srednik, Kristina Lantz, Jessica A. Hicks, Brenda R. Morningstar-Shaw, Tonya A. Mackie, Linda K. Schlater

**Affiliations:** United States Department of Agriculture, Animal and Plant Health Inspection Service, National Veterinary Services Laboratories, Ames, Iowa, United States of America; Cornell University, UNITED STATES

## Abstract

*Salmonella enterica* subspecies *enterica* serotype Dublin is a host-adapted serotype in cattle, associated with enteritis and systemic disease. The primary clinical manifestation of *Salmonella* Dublin infection in cattle, especially calves, is respiratory disease. While rare in humans, it can cause severe illness, including bacteremia, with hospitalization and death. In the United States, *S*. Dublin has become one of the most multidrug-resistant serotypes. The objective of this study was to characterize *S*. Dublin isolates from sick cattle by analyzing phenotypic and genotypic antimicrobial resistance (AMR) profiles, the presence of plasmids, and phylogenetic relationships. *S*. Dublin isolates (n = 140) were selected from submissions to the NVSL for *Salmonella* serotyping (2014–2017) from 21 states. Isolates were tested for susceptibility against 14 class-representative antimicrobial drugs. Resistance profiles were determined using the ABRicate with Resfinder and NCBI databases, AMRFinder and PointFinder. Plasmids were detected using ABRicate with PlasmidFinder. Phylogeny was determined using vSNP. We found 98% of the isolates were resistant to more than 4 antimicrobials. Only 1 isolate was pan-susceptible and had no predicted AMR genes. All *S*. Dublin isolates were susceptible to azithromycin and meropenem. They showed 96% resistance to sulfonamides, 97% to tetracyclines, 95% to aminoglycosides and 85% to beta-lactams. The most common AMR genes were: sulf2 and tetA (98.6%), aph(6)-Id (97.9%), aph(3’’)-Ib, (97.1%), floR (94.3%), and blaCMY-2 (85.7%). All quinolone resistant isolates presented mutations in *gyr*A. Ten plasmid types were identified among all isolates with IncA/C2, IncX1, and IncFII(S) being the most frequent. The *S*. Dublin isolates show low genomic genetic diversity. This study provided antimicrobial susceptibility and genomic insight into *S*. Dublin clinical isolates from cattle in the U.S. Further sequence analysis integrating food and human origin *S*. Dublin isolates may provide valuable insight on increased virulence observed in humans.

## Introduction

The CDC estimates that each year in the United States, *Salmonella enterica* causes 1.2 million infections, 24,000 hospitalizations, and 450 deaths [1]. According to FoodNet data, *Salmonella* Dublin was more commonly isolated from blood (61%) than were other *Salmonella* (5%) [1]. According to surveillance data from the National Antimicrobial Resistance Monitoring System (NARMS), the proportion of resistant isolates is higher among *S*. Dublin than among other serotypes [1]. Human outbreaks were reported in several countries [2–4]. A 2019 *S*. Dublin outbreak in the U.S. was linked to ground beef with 13 cases reported with 9 hospitalizations and 1 death in 8 states [4]. Despite the relatively low incidence of human cases, zoonotic or foodborne transmission of *S*. Dublin is of high concern because of the increased antimicrobial resistance and elevated hospitalization and death rate in humans [1]. A relatively high proportion of human infections are associated with invasive disease as a result of the acquisition of possible virulence factors such as Vi antigen [5, 6].

Salmonellosis may cause severe disease in cattle and poses a significant zoonotic risk. Farm workers, calf handlers, and their families are clearly at risk of becoming infected by *Salmonella* spp. during outbreaks of clinical illness, but the risk of exposure goes far beyond farm workers or veterinarians with direct animal contact during outbreaks of disease. Subclinical shedding of *Salmonella*, a characteristic of *Salmonella* Dublin infection, is also an issue with other common cattle serotypes such as Newport and Typhimurium, and creates risk for people in direct contact with the animal, its feces, or milk [7]. There is also risk for foodborne transmission from exposure to contaminated meat from cattle infected with *Salmonella* Dublin, including dairy beef and cull dairy cows, typically via fecal contamination of the carcass at the time of slaughter [8]. In addition to contaminated meat products, contaminated produce and unpasteurized dairy products, the long-term environmental contamination is a risk for transmission to animals and humans [1, 7].

In the United States, *Salmonella* Dublin has become one of the most multidrug-resistant (MDR) serotypes [8]. The increasing prevalence of *Salmonella* Dublin infection in the U.S. dairy industry and its unique status as host-adapted in cattle merit more specific attention [9]. A National Veterinary Services Laboratories (NVSL) *Salmonella* serotyping report from a 2017 study [10] demonstrated that of the 1,655 *Salmonella* isolates identified at the NVSL from clinical bovine case submissions, the most common serotype was *Salmonella* Dublin (26.4%), followed by *Salmonella* Cerro (17%) and *Salmonella* Montevideo (10.3%).

The antimicrobials of choice for treating bacterial gastroenteritis in humans are generally the fluoroquinolone ciprofloxacin for adults and the cephalosporin ceftriaxone for children [7].

Antimicrobial drugs are essential to protect animal health in livestock production systems [11]. *Salmonella* Dublin is a host-adapted serotype that can cause significant levels of morbidity and mortality, particularly in dairy calves, potentially necessitating antimicrobial treatment. However, the multidrug resistant nature of *S*. Dublin and limited range of approved antimicrobials often limit treatment options to supportive symptomatic treatment. If antimicrobial treatment is used without testing the susceptibility of the bacteria, treatment may be ineffective and contribute to increasing antimicrobial resistance. Because of the zoonotic implications of this disease, responsible use of antimicrobials in treatment is a critically important aspect of *S*. Dublin management, both for animal and human health [7].

The mechanism by which *S*. *enterica* typically develop antimicrobial resistance (AMR) differs according to the drug. Fluoroquinolone resistance typically occurs through clonal dissemination of *Salmonella* isolates with chromosomal mutations conferring resistance, while cephalosporin resistance usually is acquired by acquisition of mobile genetic elements via plasmids and transposons [7].

The objective of this study was to compare *Salmonella* Dublin isolates from clinical cattle samples throughout the United States during the period 2014–2017 and to analyze antimicrobial resistance profiles, presence of plasmids, and phylogenetic relationships by geographic distribution and period of time.

## Materials and methods

### Bacterial isolates

*Salmonella* Dublin diagnostic isolates from cattle (n = 140) were selected, 110 (78.5%) from clinical infections (n = 44 from lung, n = 34 from liver, n = 6 from intestine, n = 6 from feces, n = 20 from other sites) and 30 (21.4%) were of unknown clinical status, from 2014–2017 submissions for *Salmonella* serotyping archived at the NVSL. Samples came from 21 U.S. States (MN n = 37, IA n = 21, NY n = 17, SD n = 10, OH n = 7, IL n = 6, PA n = 6, TX n = 6, IN n = 5, WA n = 5, MO n = 5, KY n = 4, ID n = 2, WI n = 2, MD n = 1, AL n = 1, NE n = 1, KS n = 1, OK n = 1, MI n = 1, FL n = 1). Isolates details are available in S1 Table.

The dataset was initially limited to one sample per year per owner. If more than the targeted number of isolates remained, a randomly selected subset of isolates was chosen. The data were then de-identified to remove information other than the animal species, state of origin, clinical status, and sample type and assigned a unique identifier. Identity was confirmed using Biotyper software with an Autoflex Speed MALDI-TOF instrument (Bruker Daltonics).

### Antimicrobial susceptibility testing

All *Salmonella* isolates were tested for antimicrobial susceptibility against 14 class-representative antimicrobial agents using the Sensititre CMV4AGNF plate (Thermo Scientific), including: gentamicin (GEN), streptomycin (STR), amoxicillin/clavulanic acid (AMC), cefoxitin (FOX), ceftriaxone (CRO), meropenem (MER), sulfisoxazole (FIS), trimethoprim/sulfamethoxazole (SXT), ampicillin (AMP), chloramphenicol (CHL), ciprofloxacin (CIP), nalidixic acid (NAL), azithromycin (AZM), and tetracycline (TET). Interpretation criteria were established by the NARMS. For statistical analysis, isolates in “intermediate” category were deemed “resistant” in this study.

### Identification of antimicrobial resistant genotype

*Salmonella* Dublin isolates were subjected to whole genome sequencing (WGS) with the Illumina MiSeq platform using 2x250 paired end chemistry and the NexteraXT library preparation kit. AMR gene alleles were detected using AMRFinder [12] and ABRicate [13] with the NCBI and ResFinder [14] databases. Plasmid replicons were identified using the PlasmidFinder [15] database and ABRicate. PointFinder was used for analysis of chromosomal point mutations [16]. Isolate sequences are publicly available in the NCBI SRA BioProject PRJNA736314.

### Relationship of antimicrobial susceptibility with antimicrobial genes

Using the phenotypic results as the reference outcome, sensitivity was calculated by dividing the number of isolates that were genotypically resistant by the total number of isolates exhibiting clinical resistance phenotypes. Specificity was calculated by dividing the number of isolates that were genotypically susceptible by the total number of isolates with susceptible phenotypes.

### Phylogenetic analysis

Phylogenetic analysis was performed with vSNP (https://github.com/USDA-VS/vSNP) using *S*. Dublin strain ATTC39184 (NCBI accession CP01919.1 as a reference. This pipeline uses short read alignment to the reference using BWA-MEM [17, 18] followed by SNP (single nucleotide polymorphism) calling with FreeBayes [19]. Alignment of calls across the SNP positions are then used to build a phylogenetic tree using RAxML [20]. MLST was determined using ABRicate and the PubMLST database.

## Results

### Antimicrobial susceptibility testing

Of the 140 isolates examined, 137 (98%) were resistant to at least one antimicrobial. Of those isolates with resistance, 99% (136/137) were resistant to more than 4 antimicrobials. The most common resistance profile was: AMC, AMP, FOX, CRO, CHL, STR, SUL, TET in 76 (54.3%) isolates, and these agents represent seven antimicrobial classes. Overall, 96% and 97% of the isolates were resistant to FIS and TET, respectively, followed by 95% with streptomycin resistance and 85% with ampicillin and ceftriaxone resistance (Table 1). Thirteen isolates showed intermediate susceptibility to FOX and they were at MICs between 8 and 32 μg/mL on retest, the gene appeared to confer some level of resistance in those isolates, but did not always cross the breakpoint threshold. All of the *Salmonella* Dublin isolates were susceptible to AZM and MER. One isolate was pan-susceptible with no predicted AMR genes.

**Table 1:** Antimicrobial resistance proportions of the 140 *Salmonella* Dublin isolates in cattle from the United States.

Drug classes	Antimicrobials	Susceptible	Intermediate	Resistant
β-LACTAMS	AMOXI CLAVULANIC	21 (15.00%)	0 (0.00%)	119 (85.00%)
	AMPICILLIN	20 (15.00%)	0 (0.00%)	120 (85.71%)
	CEFOXITIN	21 (15.00%)	13 (9.26%)	106 (75.71%)
	CEFTRIAZONE	20 (14.28%)	1 (0.71%)	119 (85.00%)
	MEROPENEM	140 (100%)	0 (0.00%)	0 (0.00%)
CHLORANPHENICOLS	CHLORAPHENICOL	9 (6.42%)	0 (0.00%)	131 (93.57%)
QUINOLONES	CIPROFLOXACIN	113 (80.71%)	25 (17.85%)	2 (1.42%)
	NALIDIXIC ACID	112 (80.00%)	0 (0.00%)	28 (20.00%)
AMINOGLYCOSIDES	GENTAMICIN	135 (96.42%)	0 (0.00%)	5 (3.57%)
	STREPTOMYCIN	7 (5.00%)	0 (0.00%)	133 (95.00%)
FOLATE PATHWAY INHIBITORS	SULFISOXAZOLE	5 (3.57%)	0 (0.00%)	135 (96.42%)
	TRIMETHOPRIM SULFA	130 (92.85%)	0 (0.00%)	10 (7.14%)
TETRACYCLINES	TETRACYCLINE	4 (2.85%)	0 (0.00%)	136 (97.14%)
MACROLIDES	AZITHROMYCIN	140 (100%)	0 (0.00%)	0 (0.000%)

### Antimicrobial resistance genes

The most commonly found antimicrobial resistance genes (ARG) both detected in 98.57% isolates were sul2 and tetA, conferring resistant to sulfonamides and tetracycline, respectively. Other frequently identified genes were aph(6)-Id (n = 137; 97.86%) and aph(3’’)-Ib (n = 136; 97.14%) genes. The floR gene was the most commonly identified gene conferring resistant to chloramphenicols (n = 132; 94.28%). BlaCMY-2 was also frequently present and confers resistance to all beta-lactams (n = 120; 85.71%) (Table 2). Resistance genes for macrolides or meropenem were not found.

**Table 2:** Antimicrobial resistance genes of 140 *Salmonella* Dublin isolated from cattle.

Drug classes	Resistance genes	Number of isolates	Positive rates (%)
β-LACTAMS	blaCMY-2	120	85.71
	blaCMY-130	1	0.71
	blaCMY-132	1	0.71
	blaTEM-1A	1	0.71
	blaTEM-1B	68	46.57
	blaTEM-150	1	0.71
PHENICOLS	floR	132	94.28
	cmlA5	4	2.85
QUINOLONES	qnrB19	2	1.42
AMINOGLYCOSIDES	ant(2’’)-Ia	5	3.57
	aph(3’’)-Ib	136	97.14
	aph(6)-Id (strB)	137	97.86
	aph(3’)-Ia	50	35.71
FOLATE PATHWAY INHIBITORS	sul1	4	2.85
	sul2	138	98.57
TETRACYCLINES	tetA	138	98.57

In addition, two ARGs were found only in the Resfinder database but not in the NCBI/AMRFinder. These genes, mdfA and aac(6’)-Iaa, were detected in all isolates using the Resfinder database (n = 140; 100%).

The most frequent AMR gene profile was: blaCMY-2, floR, aph(3’’)-Ib, aph(6)-Id, sul2, tetA in 42 (30%) isolates.

### Point mutations

**Fig 1:**
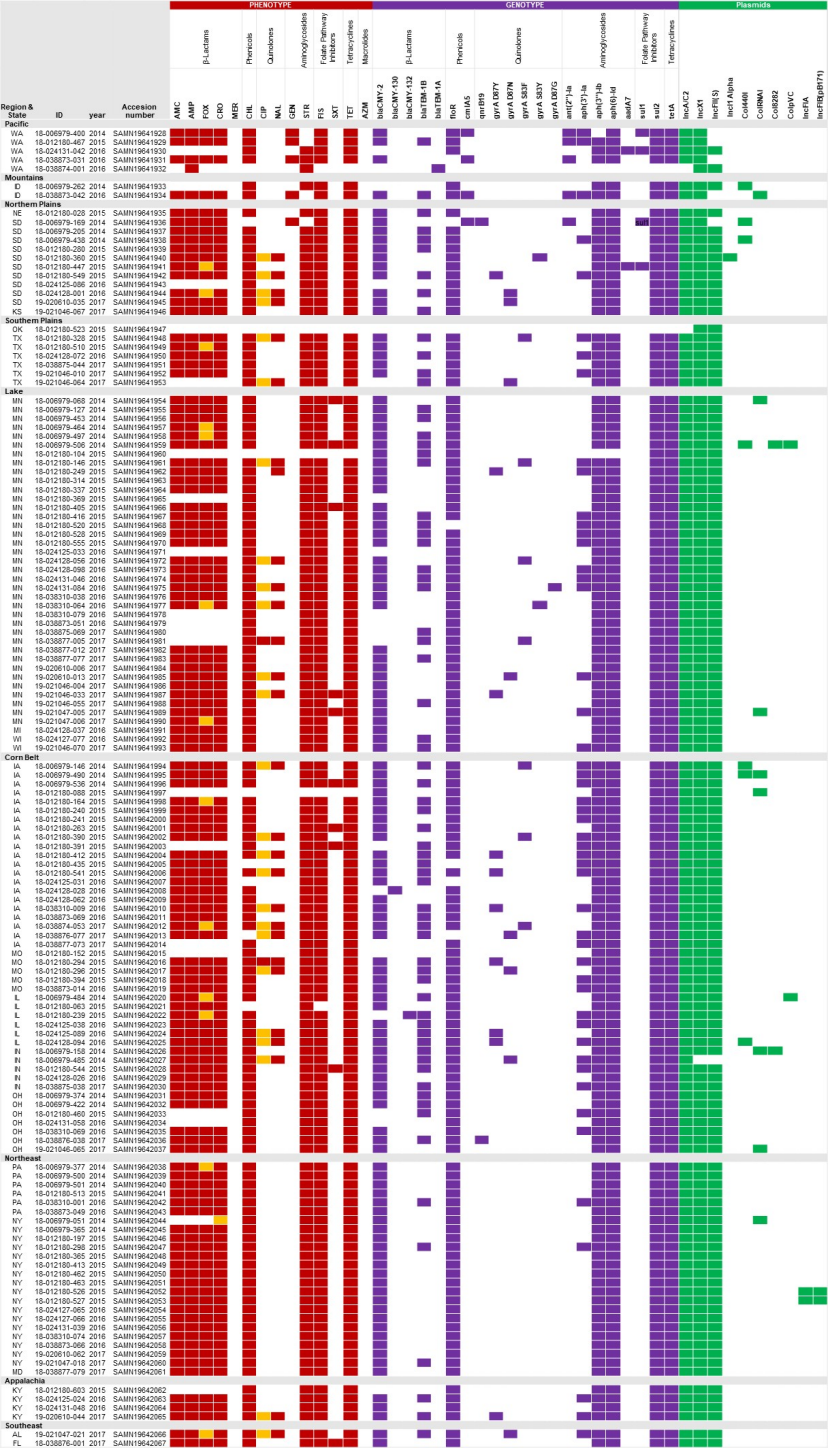
Antimicrobial susceptibility, distribution of ARGs, mutations, and presence of plasmids from the *S*. Dublin isolates in this study by state and region. Red: resistant, Orange: Intermediate, Violet: presence of gene or mutation, Green: presence of plasmid.

For ciprofloxacin, only two isolates showed phenotypic resistance and most of them (n = 25, 17.85%) exhibited weak drug resistance (intermediate) to ciprofloxacin; all these isolates (n = 27) presented chromosomal structural gene mutations in the gyrA gene (Fig 1). For nalidixic acid, 28 (20.00%) isolates exhibited phenotypic resistance with a gyrA gene mutation present. All isolates with quinolone point mutation (n = 28) exhibited a single mutation in gyrA at gyrA[D87Y] (35.7%), gyrA[D87N] (28.6%), gyrA[S83F] (25%), gyrA[S83Y] (7.1%) or gyrA[D87G] (3.6%).

### Plasmid typing

Ten plasmid types were identified among all isolates (Fig 1): IncA/C2, IncX1, IncFII(S), IncI1 Alfa, Col440I CoIRNAI, Col8282, ColpVC, IncFIA, IncFIB(pB171).

In our study, all isolates contained two to five different plasmid types with the most common ones being: IncX1 (n = 139, 99.3%), IncA/C2 (n = 138, 98.6%) and IncFII (n = 135, 96.4%).

Multi-drug resistance IncA/C2 plasmid was not found in 2 isolates. One was the pansusceptible isolate, and the second was the isolate resistant only to ampicillin with the presence of the bla-TEM gene. Fig 1 shows the antimicrobial susceptibility, distribution of ARGs, mutations, and presence of plasmids from the *S*. Dublin isolates in this study.

Geographic differences were observed for AMR genotypic and phenotypic characteristics (Fig 1). All isolates from New York were susceptible to AZM, CIP, GEN, MER, NAL and SXT; and no AMR genes or chromosomal mutations associated for those antimicrobial drugs were found. 94.1% (16/17) New York isolates were resistant to AMC, AMP, FOX, COX, CHL, STR, FIS and TET with their corresponding resistance genes present.

Annual trends in antimicrobial resistance (2014, 2015, 2016, and 2017) showed no consistent variation overall, but in two states resistance to quinolones showed a slight increase in gyrA mutation.

Isolates from MN showed an increase of resistance to quinolones by mutation in gyrA, from 0% (n = 0 isolates) in 2014, 5.4% (n = 2 isolates) in 2015, 8.1% (n = 3 isolates) in 2016, and 8.1% (n = 3 isolates) in 2017.

In SD, there was also an increase of quinolone resistance. In 2014, the resistance was conferred by qnrB19 for nalidixic acid. In 2015 resistance to nalidixic acid was conferred by mutation in gyrA. We also can observe a change of resistance for ciprofloxacin from 0% (n = 0 isolates) in 2014, 20% (n = 2 isolates) in 2015, 10% (n = 1 isolate) in 2016, and 10% (n = 1 isolate) in 2017.

### Relationship of antimicrobial susceptibility with antimicrobial genes

Table 3 shows the correlation between phenotype and genotype results. Columns 2 and 3 shows the false positives or false negatives with the phenotype method. A subset of isolates was resistant to trimethoprim sulfamethoxazole, but did not present with any known genetic mechanism of resistance.

**Table 3:** Relationship of antimicrobial susceptibility with antimicrobial resistance genes.

	Phenotype: Resistant (R)		Phenotype: Susceptible (S)	
Drug classes	Genotype: R	Genotype: S	Genotype: R	Genotype: S
β-lactams				
AMP (Penicillin)	120	0	7	13
AMC (β-lactam/β-lactamase inhibitor)	119	0	3	18
FOX (Cephems)	119	0	4	17
CRO (Cephems)	120	0	2	18
Phenicols				
CHL	131	0	3	6
Quinolones				
CIP	27	0	3	110
NAL	28	0	2	110
Aminoglycosides				
GEN	5	0	0	135
STR	132	1	5	2
Folate pathway inhibitors				
FIS	135	0	3	2
SXT	0	10	0	130
Tetracycline				
TET	136	0	2	2
Macrolides				
AZM	0	0	0	140

### Phylogenetic relationships

**Fig 2:**
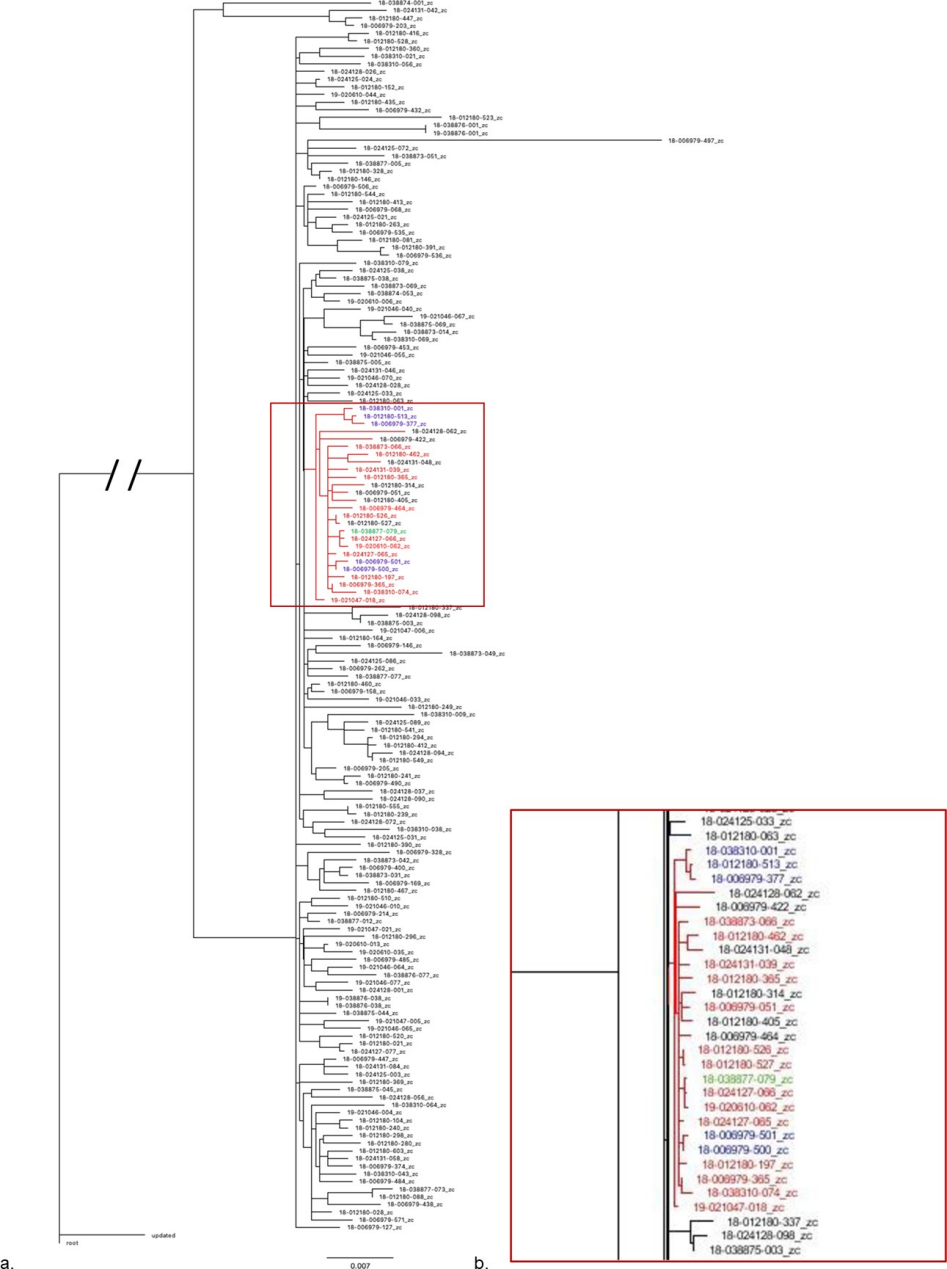
Phylogenetic tree of *S*. Dublin isolates. a) All isolates. b) NY isolates (in red) showing a common relationship on a single branch. Other isolates from northest region are colored (PA isolates in blue and MD isolates in green).

*Salmonella* Dublin isolates are highly clonal. The majority of isolates in this study fall into a single clade (Fig 2A) with a small number of isolates representing a distinct but closely related branch (Fig 2B).

All isolates except one were classified into one sequence type (ST), ST10, based on multi-locus sequence type (MLST) analysis from genome sequence*s*. One isolate presented a new PurE allele, which has not currently been assigned a new MLST sequence type.

## Discussion

*Salmonella* Dublin has developed into one of the most antimicrobial-resistant serotypes in the United State*s*. A recent study from the FDA [9], showed that among *Salmonella* Dublin isolates recovered from sick cattle and retail meats in Arizona, all except 1 were resistant to more than 4 antimicrobial classes tested. The results of our study are consistent with the phenotypic resistance profile when the dataset is extended to a nationally distributed sampling.

In our study, the predominant gene conferring beta-lactam resistance was blaCMY-2 (85.7%) followed by blaTEM-1 (47%). Hsu et al. [9], and other studies from different countries also showed that beta-lactam resistance is driven largely by the presence of blaCMY-2 [21]. Another study which compared non-typhoidal *Salmonella* isolates from humans and retail meat from FDA [22], showed that among beta-lactam-resistant strains, blaCTX-M-14b, blaFOX-6, blaLAP-1, and blaOXA were found only in human isolates and blaSHV-2a and CTX-M-1 were found in retail meat isolates. This suggests that phenotypic resistance patterns in a foodborne pathogen do not necessarily correspond to risk of zoonotic transmission; and therefore, phylogenetic analysis and identification of the specific AMR genes found in animal populations and comparison to sequence data from foodborne outbreaks has potential to provide greater insight into zoonotic transmission.

Multiple genes and mechanisms have been shown to cause quinolone resistance, Mangat et al. [21], showed that in ciprofloxacin and nalidixic acid non-susceptible isolates, an altered chromosomal gyrA gene was detected. Some quinolone resistance-determining gyrase A SNPs found in our *S*. Dublin isolates were found in other studies; two gyrA SNPs (gyrA D87N and gyrA S83F) [9] and three gyrA SNPs (D87G, S83F, D87Y) [21]. In our study, all isolates with a gyrA mutation presented reduced susceptibility to ciprofloxacin, a similar finding to Mangat et al. [21] results from animal sourced isolates. This mutation is frequently found in many *Salmonella* serotypes, and has been associated with clinical resistance [23]. A single mutation in gyrA has been associated with reduced susceptibility to ciprofloxacin and double or triple mutations have been associated with resistance to ciprofloxacin in other *Salmonella* serotypes [22, 24]. However, the presence of the qnrB19 gene has also been described in other serotypes with no corresponding phenotypic resistance [24]. When using WGS to detect genes or mutations related to quinolone resistance in *Salmonella*, the results must be interpreted with caution because they do not always correlate to clinical antimicrobial resistance. McDermott et al. [22] in the comparison study with *Salmonella* isolated from retail meat and humans, found quinolone resistance mechanisms (plasmid mediated genes or mutations) in human isolates but not in retail meat isolates with all of those isolates phenotypically susceptible to quinolones.

In aminoglycoside resistant isolates, aph(3’’)-Ib and aph(6)-Id genes were also predominantly found in other studies [25, 26]. Additionally, these 2 genes are usually found together in a HI type plasmid [27].

Two AMR genes were found in all isolates using only the Resfinder database. The mdfA gene is a multidrug efflux pump that confers resistance to lipophilic compounds (tetracycline, rifampicin, puromycin) and also confers resistance to chloramphenicol, erythromycin, fluoroquinolones (ciprofloxacin and norfloxacin) and to a much lesser extent, to certain aminoglycosides (neomycin and kanamycin) [28]. The mdfA displays a remarkably broad spectrum of drug recognition. MdfA-associated resistance to aminoglycosides is reproducible, but resistance level is very low and further studies are needed to demonstrate that these drugs are truly exported by mdfA [29]. The level of resistance may be related to the level of expression of MdfA, and the amount of MdfA in the cells is likely not very high. This gene does not appear to correlate to significant resistance in our isolates because this gene was present in all isolates, including the two more susceptible isolates.

The aac(6’)-Iaa gene confers high-level resistance to tobramycin and kanamycin, as well as a significantly increased resistance to amikacin [30]. In the susceptibility determination by microbroth dilution (Sensititre, Thermo Scientific) used in this study, only gentamicin and streptomycin were tested; thus we cannot determine if the gene was expressed.

We found some discordance in the association of genotypic and phenotypic data, as in other studies [31]. The presence of a resistance gene does not necessarily confer phenotypic resistance, and the absence of resistance genes does not unequivocally determine the phenotypic susceptibility. The phenomenon of AMR, then, is not just related to the mere presence or absence of resistance gene*s*. Other mechanisms such as enzyme activation, target modification/protection, regulation of AMR gene expression, or even change in the cell wall charge also play important roles in AMR. We observed that some isolates were resistant to trimethoprim sulfamethoxazole, but they did not present any known resistance gene. This could be due to a promoter, frameshift, or point mutation, for example [32]. Another aspect of a potentiated or combination drug is that resistance may be more multifactorial and less simple to determine genetically.

IncA/C2, IncX1 and IncFII are the most prevalent plasmids present in *S*. Dublin, as in other U.S. studies [9, 33]. IncA/C2 is a plasmid associated with MDR, and IncX1-IncFII is associated with virulence. Hsu et al. [9] showed that the IncA/C2 MDR plasmid is commonly present in *S*. Dublin and often carries many ARGs, including blaCMY-2 (65.2%). In our study 85.7% of isolates (n = 120) were positive for blaCMY-2 gene, and also carried the IncA/C2 plasmid.

In Canada, Mangat et al. [21] showed that MDR isolates often had an IncA/C2 plasmid. They reported a rise in MDR among *S*. Dublin isolates in Canada isolates from 2003 to 2015, they showed that multidrug-resistance was driven by the presence of IncA/C2 plasmids. Before 2009, *S*. Dublin isolates from animals were pansusceptible carrying only virulence plasmids (IncX1 and IncFII(S)), and after 2010 they showed that all *S*. Dublin isolates from animals were resistant to more than three drugs classes and all these isolates except one were carrying the IncA/C2 plasmids. Our results are in concordance with this report: only 2 isolates lacked IncA/C2 gene, one of these did not present any AMR genes, and the other only presented the bla-TEM gene. The carriage of IncA/C2 plasmid was seen as a typical feature of *S*. Dublin isolates from the bovine hosts in China [31], with similar resistance patterns in isolates over various years (2007 to 2012).

Virulence plasmid IncX1 was the most prevalent plasmid among our isolates (99.3%); and in a previous study in the U. S. [33], the IncX1 plasmid was detected in all *S*. Dublin genome*s*. This is noteworthy because this plasmid is not detected frequently in other serotypes, including *S*. Typhimurium and *S*. Newport. In the same study [33], Carroll et al. compared salmonellae from dairy cattle and humans in New York State (NY) and Washington State (WA); and geographic differences were observed for AMR genotypic and phenotypic characteristics in isolates from those two states. The IncFII(S) plasmid was more commonly detected in isolates from NY State. This differs from our results where IncFII(S) was the most prevalent plasmid among WA isolates and all NY isolates had IncA/C2, IncFII(S) and IncX1 plasmids in the same proportion. Another interesting result found in the Carroll et al. study was the presence of an allele on a truncated strA gene in isolates from the WA State clade which appeared to not confer STR resistance, while still being identified computationally as an STR resistance determinant. Our results showed similar AMR phenotypic and genotypic characteristics in NY isolates; but we could not observe similar characteristics for WA, potentially because our sample size was not very large for this state (only 5 isolates). The seeming conflict in these results emphasize that convenience sampling of submitted isolates comes with inherent sampling bias and may not present a full picture of antimicrobial resistance. Longitudinal studies with collection of data on management practices and antimicrobial use would be beneficial for obtaining a clearer picture of the movement of isolates and antimicrobial resistance genes and potentially aiding in the development of prevention and intervention strategies.

Mangat et al. [21] found a close relationship with U.S. isolates which showed similar circulating plasmids and mobile elements. Interestingly, the network of MDR isolates was comprised of both human and bovine isolates, whereas the network of susceptible isolates was primarily from human sources. A study in China [31] also showed that MDR was higher in animal isolates than in human ones. The presence of higher rates of MDR in animals as compared to human isolates may indicate that the requirement for more intensive management of *S*. Dublin in cattle including antibiotic use in treatment of the severe disease that *S*. Dublin can cause in calves may have been a driver for antimicrobial resistance in this serotype. However, the emergence of MDR strains in other serotypes often correspond to more complex host-pathogen interactions and genetic factors unrelated to the actual antimicrobial resistance genes [34, 35]. Nonetheless, the presence of high levels of MDR in this serotype suggest that at this time improved management, prevention and intensive supportive treatment may present a more sustainable method for approaching this disease. Use of antimicrobials should be approached cautiously and be informed by antimicrobial susceptibility test information.

All *S*. Dublin isolates are usually identified by MLST as ST10 in most of the studies from different countries [21, 31]. Our results showed ST10 for all isolates except one which was a new type, in concordance with a study in the United Kingdom [36]. A single sequence type is correlated with a highly conserved serotype such as *S*. Dublin. The vSNP results provide a much higher resolution platform for looking at single SNP changes in the bacteria, creating the potential to look at transmission dynamics and even potentially movement of ARGs on a much finer level. In addition, we could observe that several SNPs were present across multiple isolates that did not otherwise correspond with the phylogenetic relationships; and when we investigated those further, they were mutations associated with antimicrobial resistance. A highly clonal relationship among *S*. Dublin isolates has been reported [9]. The lack of genetic diversity in *S*. Dublin can be largely explained by its unique status as host adapted in cattle. Isolates from New York show an even more conserved population, with 82.4% of isolates of New York origin in the same branch. Some isolates in this branch differ by only 2 SNPs. This may be because of selection pressure or it may represent a stable population of cattle with less movement and transfer of bacteria with other geographic regions. We can observe that NY isolates have a similar ARG profile as well.

While our dataset represents a more geographically diverse sample set of clinical isolates than previous veterinary studies in the United States, expansion of the analysis to include comparison to human- and food-associated isolates from a comparable time period may help to better understand transmission dynamics and the relationship between pathogenic circulating isolates in cattle and those capable of causing significant human disease. In addition, this sample set represents submissions for diagnosis of disease; and therefore, may not be representative of the population of *S*. Dublin strains circulating in normal healthy cattle. The use of longitudinal studies with concurrent data collection for management practices and antimicrobial use have the potential to provide a much more complete picture of the organism in cattle. A better understanding of the relationship between these populations would help to target intervention strategies at the farm level to *Salmonella* strains that are more likely to cause animal or human disease, allowing for effective targeted intervention.

## Supporting information

S1 Table Isolates details (sample ID, year of isolation, clinical rol, sample source, owner state, accession number, antimicrobial susceptibility test results). (XLSX)

## References

[1] Harvey RR, Friedman CR, Crim SM, Judd M, Barrett KA, Tolar B, et al. Epidemiology of *Salmonella enterica* Serotype Dublin Infections among Humans, United States, 1968–2013. Emerg Infect Dis. 2017;23(9):1493–1501. doi: 10.3201/eid2309.170136 28820133 PMC5572876

[2] Cadel-Six S, Vignaud ML, Mohammed M. Draft Genome Sequences of *Salmonella* enterica subsp. enterica Serovar Dublin Strains from St. Nectaire and Morbier Cheeses Characterized by Multilocus Variable-Number Tandem-Repeat Analysis Profiles Associated with Two Fatal Outbreaks in France. Microbiol Resour Announc. 2019 Jan 3;8(1):e01361–18. doi: 10.1128/MRA.01361-18 Erratum in: Microbiol Resour Announc. 2020 Feb 13;9(7): PMCID: PMC6318359. 30637388 PMC6318359

[3] Mohammed M, Thapa S. Evaluation of WGS-subtyping methods for epidemiological surveillance of foodborne salmonellosis. One Health Outlook. 2020;2(13). doi: 10.1186/s42522-020-00016-5 33829134 PMC7993512

[4] CDC Centers for Disease Control and Prevention. Outbreak of Salmonella Infections Linked to Ground Beef. https://www.cdc.gov/salmonella/dublin-11-19/index.html.

[5] Mohammed M, Cormican M. Whole genome sequencing provides insights into the genetic determinants of invasiveness in *Salmonella* Dublin. Epidemiol Infect. 2016;144(11):2430–2439. doi: 10.1017/S0950268816000492 26996313 PMC9150539

[6] Mohammed M, Vignaud ML, Cadel-Six S. Whole-Genome Sequences of Two *Salmonella enterica* Serovar Dublin Strains That Harbour the *viaA*, *viaB*, and *ompB* Loci of the Vi Antigen. Microbiol Resour Announc. 2019;8(4):e00028–19. doi: 10.1128/MRA.00028-19 30948462 PMC6449553

[7] Holschbach CL, Peek SF. *Salmonella* in Dairy Cattle. Vet Clin North Am Food Anim Pract. 2018; 34(1):133–154. doi: 10.1016/j.cvfa.2017.10.005 29224803 PMC7135009

[8] Mc Donough PL, Fogelman D, Shim SJ, Brunner MA, Lein DH. *Salmonella* enterica serotype Dublin infection: an emerging infectious disease for the northeastern United States. J Clin Microbiol. 1999; 37(8):2418–2427. doi: 10.1128/JCM.37.8.2418-2427.1999 10405378 PMC85243

[9] Hsu CH, Li C, Hoffmann M, McDermott P, Abbott J, Ayers S, et al. Comparative Genomic Analysis of Virulence, Antimicrobial Resistance, and Plasmid Profiles of *Salmonella* Dublin Isolated from Sick Cattle, Retail Beef, and Humans in the United States. Microb Drug Resist. 2019;25(8):1238–1249. doi: 10.1089/mdr.2019.0045 31149890 PMC11555760

[10] Morningstar-Shaw, BR. National Veterinary Services Laboratories (NVSL) *Salmonella* serotyping report. Proceedings 122nd Annual Meeting US Animal Health Association, Kansas. 2018 Oct 18–24. pp. 280–283. Available from: https://usaha.org/upload/Proceedings/2018_Proceedings_v2_Comb_FINAL.pdf

[11] Caudell MA, Dorado-Garcia A, Eckford S, Creese C, Byarugaba DK, Afakye K, et al. Towards a bottom-up understanding of antimicrobial use and resistance on the farm: A knowledge, attitudes, and practices survey across livestock systems in five African countries. Plos One. 2020;15(1):e0220274. doi: 10.1371/journal.pone.0220274 31978098 PMC6980545

[12] Feldgarden M, Brover V, Haft DH, Prasad AB, Slotta DJ, Tolstoy I, et al. Validating the AMRFinder Tool and Resistance Gene Database by Using Antimicrobial Resistance Genotype-Phenotype Correlations in a Collection of Isolates. Antimicrob Agents Chemother. 2019 Oct 22;63(11):e00483–19. doi: 10.1128/AAC.00483-19 Erratum in: Antimicrob Agents Chemother. 2020 Mar 24: PMCID: PMC6811410. 31427293 PMC6811410

[13] Seemann T. Abricate, Github. Available from https://github.com/tseeman/abricate/.

[14] Zankari E, Hasman H, Cosentino S, Vestergaard M, Rasmussen S, Lund O, et al. Identification of acquired antimicrobial resistance genes. J Antimicrob Chemother. 2012;67(11):2640–2644. doi: 10.1093/jac/dks261 22782487 PMC3468078

[15] Carattoli A, Zankari E, García-Fernández A, Voldby Larsen M, Lund O, Villa L, et al. In silico detection and typing of plasmids using PlasmidFinder and plasmid multilocus sequence typing. Antimicrob Agents Chemother. 2014;58(7):3895–3903. doi: 10.1128/AAC.02412-14 24777092 PMC4068535

[16] Zankari E, Allesøe R, Joensen KG, Cavaco LM, Lund O, Aarestrup FM. PointFinder: a novel web tool for WGS-based detection of antimicrobial resistance associated with chromosomal point mutations in bacterial pathogens. J Antimicrob Chemother. 2017; 72(10):2764–2768. doi: 10.1093/jac/dkx217 29091202 PMC5890747

[17] Li H. Aligning sequence reads, clone sequences and assembly contigs with BWA-MEM. arXiv:1303.3997v1 [Preprint]. 2013 [cited 2013 May 26]. Available from: https://arxiv.org/abs/1303.3997v2

[18] Vasimuddin Md, Misra Sanchit, Li Heng, Aluru Srinivas. Efficient Architecture-Aware Acceleration of BWA-MEM for Multicore Systems. IEEE Parallel and Distributed Processing Symposium (IPDPS), 2019.

[19] Garrison E, Marth G. Haplotype-based variant detection from short-read sequencing. arXiv:1207.3907 [Preprint]. 2012 [cited 2012 Jul 20]. Available from: https://arxiv.org/abs/1207.3907

[20] Stamatakis A. RAxML version 8: A tool for Phylogenetic Analysis and Post-Analysis of Large Phylogenies. Bioinformatics. 2014;30(9):1312–1313. doi: 10.1093/bioinformatics/btu033 24451623 PMC3998144

[21] Mangat CS, Bekal S, Avery BP, Côté G, Daignault D, Doualla-Bell F, et al. Genomic investigation of the emergence of invasive multidrug resistant *Salmonella* Dublin in humans and animals in Canada. Antimicrob Agents Chemother. 2019;63(6):e00108–19. doi: 10.1128/AAC.00108-19 31036694 PMC6535508

[22] McDermontt PF, Tyson GH, Kabera C, Chen Y, Li C, Folster JP, et al. Whole-Genome Sequencing of detecting Antimicrobial Resistance in Nontyphoidal *Salmonella*. Antimicrob Agents Chemother. 2016; 60(9):5515–5520. doi: 10.1128/AAC.01030-16 27381390 PMC4997858

[23] Lee HY, Su LH, Tsai MH, Kim SW, Chang HH, Jung SI, et al. High rate of reduced susceptibility to ciprofloxacin and ceftriaxone among nontyphoid Salmonella clinical isolates in Asia. Antimicrob Agents Chemother. 2009 Jun;53(6):2696–9. doi: 10.1128/AAC.01297-08 19332677 PMC2687261

[24] Day MR, Doumith M, Do Nascimento V, Nair S, Ashton PM, Jenkins C, et al. Comparison of phenotypic and WGS-derived antimicrobial resistance profiles of *Salmonella enterica* serovars Typhi and Paratyphi, J Antimicrob Chemother, 2018;73(2):365–372. doi: 10.1093/jac/dkx379 29216342

[25] Yasit M, Farman M, Shah MW, Jiman-Fatani AA, Othman NA, Almasaudi SB, et al. Genomic and antimicrobial resistance genes diversity in multidrug-resistant CTX-M-positive isolates of *Escherichia coli* at a health care facility in Jeddah. J Infect Public Health. 2020;13:94–100. doi: 10.1016/j.jiph.2019.06.011 31279801

[26] Cohen E, Davidovich M, Rokney A, Valinsky L, Rahav G, Gal-Mor O. Emerge of new variant of antibiotic resistance islands among multidrug-resistant *Salmonella enterica* in poultry. Environ Microbiol. 2019;22(1):413–432. doi: 10.1111/1462-2920.14858 31715658

[27] MacMillan EA, Gupta SK, Williams LE, Jove T, Hiott LM, Woodley TA, et al. Antimicrobial Resistance genes, cassettes, and plasmids present in *Salmonella enterica* associated with United States food animals. Front Microbiol. 2019;10:832. doi: 10.3389/fmicb.2019.00832 31057528 PMC6479191

[28] Adzitey F, Assoah-Peprah P, Ayum TG. Whole-genome sequencing of *Eschrichia coli* isolated from contaminated meat samples collected from the Northern region of Ghana reveals the presence of multiple antimicrobial resistance genes. J Glob Antimicrob Resist. 2019;18:179–182. doi: 10.1016/j.jgar.2019.03.014 30926467

[29] Edgar R, Bibi E. MdfA, an Escherichia coli multidrug resistance protein with an extraordinarily broad spectrum of drug recognition. J Bacteriol. 1997 Apr;179(7):2274–80. doi: 10.1128/jb.179.7.2274-2280.1997 Erratum in: J Bacteriol 1997 Sep;179(17):5654. PMCID: PMC178964. 9079913 PMC178964

[30] Salispante S, Hall B. Determining the limits of the evolutionary potential of an antibiotic resistance gene. Mol Biol Evol, 2003;20(4):653–659. doi: 10.1093/molbev/msg074 12679553

[31] Paudyal N, Pan H, Elbediwi M, Zhou X, Peng X, Li X, et al. Characterization of *Salmonella* Dublin isolated from bovine and human hosts. BMC Microbiol. 2019;19(1):226. doi: 10.1186/s12866-019-1598-0 31619165 PMC6796477

[32] Nurjadi D, Zizmann E, Chanthalangsy Q, Heeg K, Boutin S. Integrative Analysis of Whole Genome Sequencing and Phenotypic Resistance Toward Prediction of Trimethoprim-Sulfamethoxazole Resistance in *Staphylococcus aureus*. Front Microbiol. 2021;11:607842. doi: 10.3389/fmicb.2020.607842 33519755 PMC7840696

[33] Carroll LM, Wiedmann M, den Bakker H, Siler J. Whole-Genome Sequencing of Drug- Resistant *Salmonella enterica* Isolates from Dairy Cattle and Humans in New York and Washington States Reveals Source and Geographic Associations. Appl Environ Microbiol. 2017;83(12):e00140–17. doi: 10.1128/AEM.00140-17 28389536 PMC5452826

[34] Bearson BL, Trachsel JM, Shippy DC, Sivasankaran SK, Kerr BJ, Loving CL, et al. The Role of *Salmonella* Genomic Island 4 in Metal Tolerance of *Salmonella enterica* Serovar I 4,[5],12:i:- Pork Outbreak Isolate USDA15WA-1. Genes (Basel). 2020 Oct 30;11(11):1291. doi: 10.3390/genes11111291 33142960 PMC7716197

[35] Tyson GH, Li C, Harrison LB, Martin G, Hsu CH, Tate H, et al. A Multidrug-Resistant *Salmonella* Infantis Clone is Spreading and Recombining in the United States. Microb Drug Resist. 2021 Jun;27(6):792–799. doi: 10.1089/mdr.2020.0389 33232624 PMC11555764

[36] Mohammed M, Le Hello S, Leekitcharoenphon P, Hendriksen R. The invasome of *Salmonella* Dublin as revealed by whole genome sequencing. The invasome of *Salmonella* Dublin as revealed by whole genome sequencing. BMC Infect Dis. 2017;17(1):544. doi: 10.1186/s12879-017-2628-x 28778189 PMC5544996

